# DB-YOLO: A Duplicate Bilateral YOLO Network for Multi-Scale Ship Detection in SAR Images

**DOI:** 10.3390/s21238146

**Published:** 2021-12-06

**Authors:** Haozhen Zhu, Yao Xie, Huihui Huang, Chen Jing, Yingjiao Rong, Changyuan Wang

**Affiliations:** 1 Institute of Microelectronics, Chinese Academy of Sciences, Beijing 100029, China; zhuhaozhen@ime.ac.cn (H.Z.); xieyao@ime.ac.cn (Y.X.); huanghuihui@ime.ac.cn (H.H.); jingchen@ime.ac.cn (C.J.); 2 University of Chinese Academy of Sciences, Beijing 100049, China; 3 Science and Technology on Near Surface Detection Laboratory, Wuxi 214035, China; enjoy_rong@163.com

**Keywords:** synthetic aperture radar (SAR), deep learning, duplicate bilateral feature pyramid network (DB-FPN), multiscale ship detection

## Abstract

With the wide application of convolutional neural networks (CNNs), a variety of ship detection methods based on CNNs in synthetic aperture radar (SAR) images were proposed, but there are still two main challenges: (1) Ship detection requires high real-time performance, and a certain detection speed should be ensured while improving accuracy; (2) The diversity of ships in SAR images requires more powerful multi-scale detectors. To address these issues, a SAR ship detector called Duplicate Bilateral YOLO (DB-YOLO) is proposed in this paper, which is composed of a Feature Extraction Network (FEN), Duplicate Bilateral Feature Pyramid Network (DB-FPN) and Detection Network (DN). Firstly, a single-stage network is used to meet the need of real-time detection, and the cross stage partial (CSP) block is used to reduce the redundant parameters. Secondly, DB-FPN is designed to enhance the fusion of semantic and spatial information. In view of the ships in SAR image are mainly distributed with small-scale targets, the distribution of parameters and computation values between FEN and DB-FPN in different feature layers is redistributed to solve the multi-scale detection. Finally, the bounding boxes and confidence scores are given through the detection head of YOLO. In order to evaluate the effectiveness and robustness of DB-YOLO, comparative experiments with the other six state-of-the-art methods (Faster R-CNN, Cascade R-CNN, Libra R-CNN, FCOS, CenterNet and YOLOv5s) on two SAR ship datasets, i.e., SSDD and HRSID, are performed. The experimental results show that the AP50 of DB-YOLO reaches 97.8% on SSDD and 94.4% on HRSID, respectively. DB-YOLO meets the requirement of real-time detection (48.1 FPS) and is superior to other methods in the experiments.

## 1. Introduction

Automatic ship detection plays an important role in both civil and military fields, such as port management, fishery development supervision, and maritime rescue [1], etc. The imaging process of Synthetic Aperture Radar (SAR) is less affected by environmental factors, can detect hidden objects, and has the ability of the all-weather and all-day operation [2]. With the successful launch of SAR satellites such as TerraSAR-X, Sentinel-1 and Gaofen-3, ship detection of SAR images has become a worldwide research hotspot [3,4,5]. At the same time, with the rapid increase in the data volume of SAR images to be processed, the requirements for accuracy and real-time performance of detection algorithms are also increasing. At present, the space-borne SAR system can achieve a high resolution of less than one meter [6], and the size differences of identifiable target ships increase, which poses a higher challenge to the multi-scale detection capability of the detection algorithms.

Traditional SAR image ship detection methods are mostly based on concrete features and traditional image processing techniques, and are mainly classified through image segmentation, handcraft feature extraction and other methods for detection, such as the Constant False Alarm Rate (CFAR) [7], Histogram of Oriented Gradient (HOG) [8], Haar [9] and so on. Traditional detection methods rely on experience and have low computational speed [10]. Modern abstract-features-based methods like CNN have developed rapidly in the field of computer vision due to their powerful representation capabilities and automatic feature extraction. At present, detection methods based on CNN can be divided into two categories: two-stage detectors and single-stage detectors. Two-stage detectors such as Faster R-CNN [11], R-FCN [12] and RepPoints [13], etc., obtain higher detection accuracy by extracting a region of interest (ROI), but the larger amount of calculation makes it difficult to meet real-time detection [10]. Single-stage detectors such as SSD [14], YOLO [15] and FCOS [16], etc., complete detection tasks based on direct position regression and directly predict the coordinates and confidence of boundary boxes from the whole image, without generating ROI in the detection process. Therefore, the single-stage detectors are faster than the two-stage detectors, but the accuracy is often inferior to the two-stage detectors.

At present, the research on applying CNN to SAR ship detection has been rapidly developed. Li et al. [17] released the first open SAR ship detection dataset (SSDD) and proposed an SAR ship detection method based on improved Faster R-CNN. Pan et al. [18] proposed a SAR ship detector called MSR2N with a rotating bounding box, but the complexity of the network makes it slow. Chen et al. [19] proposed a single-stage detector that can achieve high accuracy in inshore scenes, but it is still difficult to meet the needs of real-time detection. Bao et al. [20] proposed two pretraining techniques called optical ship detector (OSD) and optical-SAR matching (OSM) to transfer the characteristics of ships in earth observations and plentiful texture features from optical images to SAR images. Some researchers used attention modules to improve the accuracy of detectors, such as Hu et al. [21] and Jiang et al. [22], but this method usually brings a large computational cost. Zhang et al. [23] designed a detector called ShipDeNet-20 based on the idea of grid division, where the detector network is composed of only 20 layers, showing high superiority in terms of speed, but the accuracy is not satisfactory. Therefore, how to improve the accuracy while maintaining real-time detection is one of the urgent problems to be solved in SAR ship detection.

In addition to maintaining the balance between speed and accuracy, how to improve multi-scale detection capability is also the bottleneck of ship detection. Different ship types of SAR images from different sources have different resolutions, resulting in nearly a thousandfold difference between the maximum and minimum pixel areas of ships in the same data set [24]. Feature fusion networks such as FPN [25], PANet [26] and BiFPN [27] can improve multi-scale target detection by fusing feature maps of different scales. Existing studies have applied the improved Feature fusion network to ship multi-scale detection. Wei et al. [28] designed a high-resolution feature pyramid network (HRFPN) to make full use of multi-scale feature maps. Guo et al. [29] used an extended feature pyramid network (EFPN) to enhance semantics information and improve the detection capability of small-scale ships. Yang et al. [30] designed a task-wise attention feature pyramid network (TAFPN) to obtain stronger semantic information and multi-scale feature maps. The above detectors have improved the multi-scale detection capabilities by improving the feature fusion network. However, for ship targets with large differences in scale, the existing feature fusion network is still difficult to meet the requirements of SAR ship detection in actual scenes.

To sum up, the following two problems in the application of deep learning methods in the automatic detection of ships need to be further improved: (1) improve the accuracy while maintaining real-time detection; (2) multi-scale objective applicability of the algorithm. Therefore, DB-YOLO was proposed. In order to ensure real-time detection, a single-stage detection method was selected as the basic network, and we reduced the parameters and calculation in a Feature Extraction Network (FEN) through a cross stage partial (CSP) [31] structure. Then, two methods are proposed to improve multi-scale detection: (1) design a new feature fusion network called Duplicate Bilateral Feature Pyramid Network (DB-FPN), which enhances the fusion of spatial information and semantic information through the duplicate structure and the bilateral fusion network; (2) optimize the distribution of parameters and calculations of C3 to C5 layers in FEN and DB-FPN, so as to strengthen the extraction ability of features in the lower layer.

The main contributions of this paper mainly include the following aspects:

(1) A CNN-based single-stage ship detector is realized, which can reach a higher standard in both accuracy and speed.
(2) FEN is designed and reduced the complexity of the network through the residual structure. At the same time, the distribution of parameters and calculations of the C3 to C5 layers were optimized in view of the fact that the size of ships in SAR images is mainly small and medium.
(3) DB-FPN is designed to improve the multi-scale detection capabilities of ships; it enhances the fusion of spatial information and semantic information and makes full use of feature maps at different locations through feature multiplexing.
(4) Compared with the other six state-of-the-art methods on SSDD [17] and HRSID [32] data sets, the results show that DB-YOLO has better effectiveness and robustness.

The rest of this paper is divided into four sections. Section 2 introduces the proposed method. In Section 3, experiments and results in two data sets are introduced to verify the effectiveness of the DB-YOLO, and it is verified in a real Sentinel-1 scenario. Finally, Section 4 come to a conclusion.

## 2. Proposed Method

**Figure 1:**
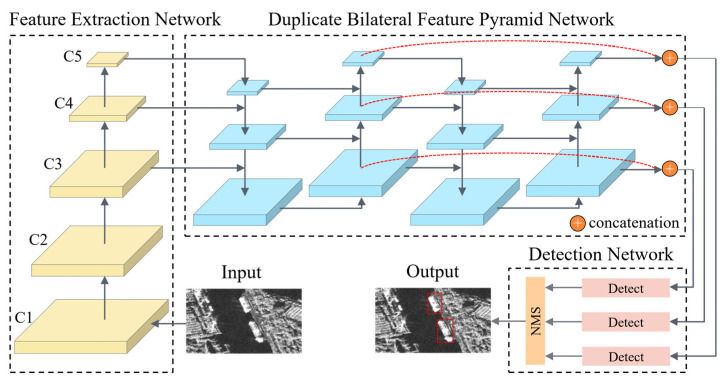
Overall architecture of the proposed method DB-YOLO. It consists of the Feature Extraction Network, Duplicate Bilateral Feature Pyramid Network and Detection Network.

The overall framework of DB-YOLO consists of three parts, as shown in Figure 1: Feature Extraction Network (FEN), Duplicate Bilateral Feature Pyramid Network (DB-FPN) and Detection Network (DN). Firstly, FEN is constructed based on the feature network of CSP block to generate multi-scale feature maps. Then, based on the multi-scale feature fusion network DB-FPN, the feature maps output by FEN are fused to obtain better spatial and semantic information. Finally, the DN can be used for classification and regression based on feature maps of different scales. The loss function is described at the end of this section.

### 2.1. Feature Extraction Network

**Figure 2:**
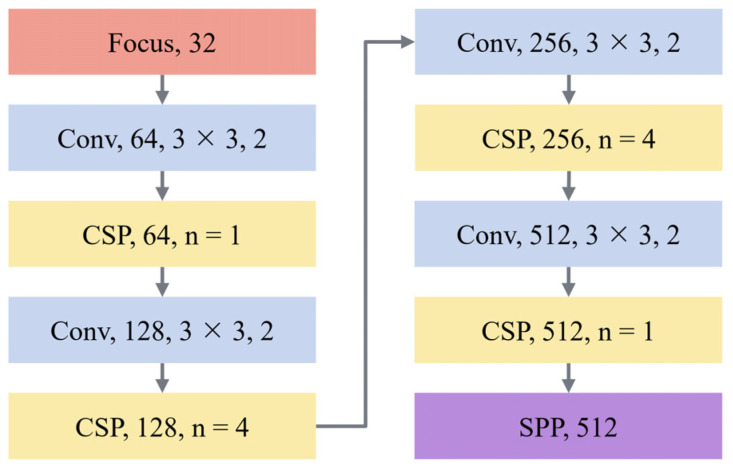
Flowchart of the Feature Extraction Network (FEN). A single block in the figure represents a single operation. The first number after the name of the block represents the channel of feature maps output by the block. The kernel and stride of the Conv block are set to 3 × 3 and 2, respectively. The n in the CSP block is the number of series-connected Res blocks.

The flowchart of FEN is shown in Figure 2. Firstly, inspired by the backbone of YOLOv5, a Focus module is added at the front of the FEN to improve the feature extraction ability and reduce the loss of information during the down-sampling process. Secondly, a convolution layer with the kernel of 3 × 3 and the step size of 2 is used for down-sampling to concentrate information in channel space. After that, features were enhanced by the CSP block, and the above steps were repeated until the size of the feature map is 1/32 of the input image. Finally, the spatial pyramid pooling (SPP) [33] module at the end of the FEN was used to increase the receptive field to improve the scale invariance of the image.

**Figure 3:**
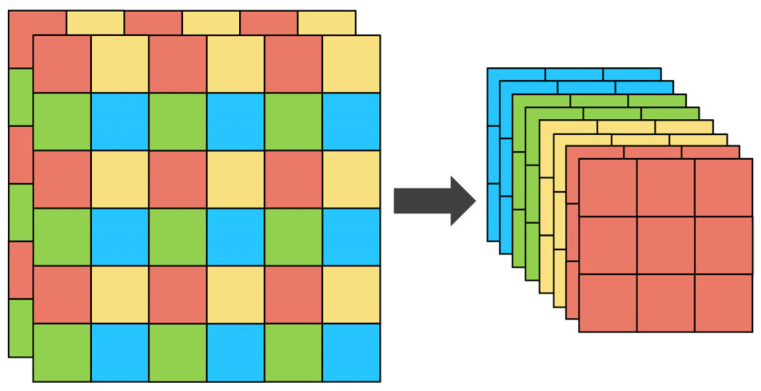
Structure of the Focus module. Left and right are the feature map before and after operation, respectively.

At the beginning of the network, the input picture is sliced through the Focus module, which splits every four adjacent pixels in a picture into four channels and concentrates the information in the channel space, thus expanding the input channel by four times. The Focus layer can avoid information loss in the process of image down-sampling and retain more complete information for subsequent feature extraction. The specific structure of the Focus module is shown in Figure 3.

The CSP block integrates the feature maps before and after the network through the residual structure, which alleviates the gradient redundancy problem of large CNNs from the perspective of network architecture. CSP block reduces the parameters and calculations through feature reuse, which not only improves the speed and accuracy but also reduces the size of the model.

**Figure 4:**
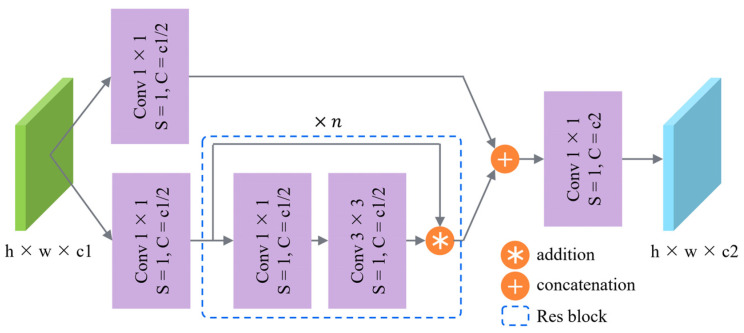
Flowchart of the CSP block. h and w mean the height and weight of the feature map, respectively. c1 means the number of input channels, and c2 means the number of output channels. S means the stride of the convolution. C means the channel of the feature map. Blue dashed box indicates the detail of Res block, and n indicates the repeated number of it.

The CSP block is divided into two parts: CSP structure and Res block. The CSP structure firstly divides the input into two branches, respectively, passing through a 1 × 1 convolution layer while reducing the number of channels to half of the input, and one of the branches passes through the Res block. Then the two branches are merged by concatenation, and finally, the number of channels is modified to the set value through a 1 × 1 convolution layer. The Res block is the residual network, whose first layer is a 1 × 1 convolution layer, and the second layer is a 3 × 3 convolution layer. Then feature maps before and after the network are fused by the residual structure. The stride of the convolution layer in the Res block is 1, and the size and channel of the input and output feature map remain unchanged. Meanwhile, in order to avoid over-fitting and enhance the nonlinear learning ability of the model, batch normalization (BN) and the activation function are added after each convolution layer. The CSP block used in this paper is shown in Figure 4.

In view of the high proportion of small ships in SAR images [32], the number of Res blocks in C3 and C4 layers is increased by strengthening the feature extraction capability in the lower layer of the FEN, while the number of Res blocks at the C5 layer that contributes less to small ship detection but needs to occupy a large amount of calculation is reduced. In this paper, the number of Res blocks in layer {C2, C3, C4, C5} is set as {1, 4, 4, 1}. At the same time, in order to further improve the utilization of the feature map and reduce redundancy, the number of channels in layer {C3, C4, C5} is set as {128, 256, 512}. In addition, since the ships in SAR image are mainly a small size, in order to prevent the loss of semantic information caused by a too deep network, C5 is seen as the highest layer in the network.

At the end of the FEN, SPP [33] is used to increase the receptive field to improve the scale invariance of the image and reduce over-fitting. SPP is independent of the CNN structure design, and it uses different sizes of kernels for max-pooling and then concatenates them in series to improve the robustness and accuracy of the network. At the same time, the SiLU activation function is used to improve the non-linear learning ability of the network and prevent falling into a local minimum during the training process.

### 2.2. Duplicate Bilateral Feature Pyramid Network

**Figure 5:**
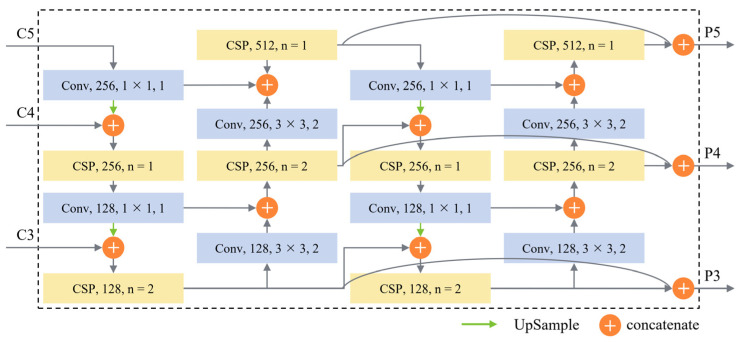
Duplicate Bilateral Feature Pyramid Network. The input is the feature map called C3–C5 in the FEN, and the output feature map called P3-P5 after fusion corresponds to it. The numbers in the figure from front to back are output channels, kernel and stride. The n in the CSP block represents the number of Res blocks.

In view of the large size variation difference of ships in high-resolution SAR images, and the existing feature fusion network is difficult to meet the needs of multi-scale ship detection, DB-FPN is proposed to obtain multi-scale feature maps with stronger semantic and spatial information. DB-FPN performs better on ships of different sizes, and the detailed structure is shown in Figure 5.

Inspired by PANet [26], the secondary fusion framework features of up-bottom and bottom-up are used in the feature fusion network in this paper. In the process of merging from top to bottom, the C5 layer in FEN first halves the number of channels through a 1 × 1 convolution layer and doubles the size of the feature map through up-sampling. Then concatenate it with the C4 layer in FEN, so the features are enhanced by the CSP block and the number of channels is halved to generate a feature map with the same size as the C4 layer. Then repeat the above process to generate a feature map of the same size as the C3 layer. In the process of bottom-up fusion, a convolution layer with the kernel of 3 × 3 and stride of 2 is used to adjust the size of the feature map, and then it is concatenated with the feature map of the same size in the up-bottom fusion process. After feature extraction by the CSP block, feature maps of the same size as the C4 layer are generated. Then repeat the above process to generate a feature map of the same size as the C5 layer. Similar to the FEN proposed above, the sensitivity of DB-FPN to small ships can be improved by increasing the number of Res blocks in the C3 and C4 layers.

Inspired by EfficientNet [27] and DetectoRS [34], duplicate architecture is designed to make the whole DB-FPN contain two series sub-networks, and each sub-network has the same structure. In order to fully reuse the parameters of the network front-end and reduce redundancy, the number of channels {C3, C4, C5} in the feature map is set to {128, 256, 512}, and finally, the outputs of each sub-network are concatenated together. Benefiting from feature multiplexing, the path between the layers of same size in the network is shortened, and the flow of information between different layers is enhanced.

### 2.3. Detection Network

In order to generate the vector of class, bounding box and confidence, in the third part of the proposed method (Detection Network, Figure 1), the detection head of YOLO [15] is used as the detection module. The feature maps of the three scales correspond to the three detection heads, respectively. The detection heads of different scales are used to detect ships of different sizes, and they are finally superimposed together. In addition, non-maximum suppression (NMS) [35] is introduced to extract bounding boxes with higher confidence so as to eliminate redundant bounding boxes.

### 2.4. Loss Function

CIoU Loss [36] is used as the loss of bounding box. Compared with other methods, such as DIoU Loss [36] and GIoU Loss [37], CIoU Loss has a faster convergence speed and better performance. The composition of the Loss function can be expressed as Equation (1):

(1) $$Loss=\lambda_{coord}L_{coord}+\lambda_{conf}L_{conf}+\lambda_{cls}L_{cls}$$

where $L_{coord}$, $L_{conf}$ and $L_{cls}$ indicates the error of coordinate, confidence and classification, while $\lambda_{coord}$, $\lambda_{conf}$ and $\lambda_{cls}$ are the weight coefficients of coordinate, confidence and classification, respectively.

**Figure 6:**
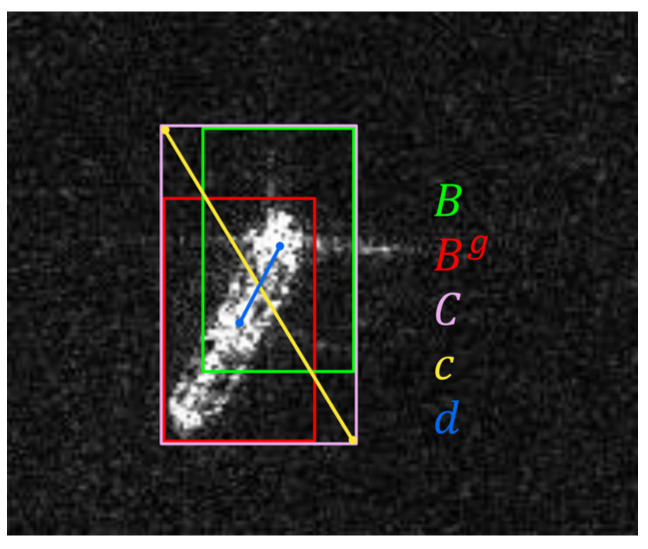
Visualization of coordinate error of *CIoU* losses. B and $B^{g}$ is the predicted bounding box and ground-truth bounding box, respectively. *C* is the smallest box covering B and $B^{g}$. *c* is the diagonal length of *C* and *d* is the distance of central points of B and $B^{g}$.

In the coordinate loss, *IoU* is usually as the metric, and its formula is as follows:

(2) $$L_{coord}=\overset{S\times S}{\underset{i=0}{\sum }}\overset{B}{\underset{j=0}{\sum }}I_{ij}^{obj}\left(1-CIoU\right)$$

(3) $$CIoU=IoU-\frac{d^{2}}{c^{2}}-\alpha v$$

(4) $$IoU=\left|\frac{B\cap B^{g}}{B\cup B^{g}}\right|$$

(5) $$\alpha =\frac{v}{\left(1-IoU\right)+v}$$

(6) $$v=\frac{4}{\pi^{2}}{\left(arctan\frac{w^{g}}{h^{g}}-arctan\frac{w}{h}\right)}^{2}$$

where $I_{ij}^{obj}$ represents the j-th anchor of the i-th cell containing the target. B is the predicted bounding box, and $B^{g}$ is the ground-truth bounding box. *C* is the smallest box covering B and $B^{g}$, and *c* is the diagonal length of *C*. *d* is the distance of central points of two boxes. *α* is a positive trade-off parameter, and *v* measures the consistency of the aspect ratio. $w^{g}$ and $h^{g}$ (*w* and *h*) are the width and height of $B^{g}$ (*B*), respectively. The visualization of coordinate error of CIoU losses is shown in Figure 6.

The confidence loss function is calculated as Equation (7):

(7) $$L_{conf}=\lambda_{obj}\overset{S\times S}{\underset{i=0}{\sum }}\overset{B}{\underset{j=0}{\sum }}I_{ij}^{obj}{\left(N-N^{g}\right)}^{2}+\lambda_{noobj}\overset{S\times S}{\underset{i=0}{\sum }}\overset{B}{\underset{j=0}{\sum }}I_{ij}^{noobj}{\left(N-N^{g}\right)}^{2}$$

where N and $N^{g}$ are the confidence scores of the prediction box and ground truth, respectively. $I_{ij}^{obj}$ and $I_{ij}^{noobj}$ indicate whether the ship in j-th anchor box of the i-th cell, respectively. $\lambda_{obj}$ and $\lambda_{noobj}$ represent the weight for whether the anchor contains the ship, respectively.

The classification loss function is calculated as Equation (8):

(8) $$L_{cls}=\overset{S\times S}{\underset{i=0}{\sum }}\overset{B}{\underset{j=0}{\sum }}I_{ij}^{obj}\left[p^{g}\left(n\right)log\left(p\left(n\right)\right)+\left(1-p^{g}\left(n\right)\right)log\left(1-p\left(n\right)\right)\right]$$

where $I_{ij}^{obj}$ represents the ship in j-th anchor of the i-th cell, *n* is the target category and p(n) and $p^{g}\left(n\right)$ are the predicted category and ground truth category, respectively.

## 3. Experiments and Results

### 3.1. Experiment Settings

The experiment environment was performed on a PC with Intel core i7-10750H CPU, GeForce RTX 2060 Max-Q (6GB storage), CUDA 11.0, CUDNN 8.0, and the PC operating system was Ubuntu 18.04 LTS. The deep learning framework was Pytorch 1.7. The optimizer used stochastic gradient descent (SGD) to train the network. The initial learning rate was 0.01, and the final learning rate was 0.002. The data augmentation method of image mosaic and image flipping were used in the initial image processing.

### 3.2. Data Sets

In the experiment stage, two SAR ship data sets (SSDD [17] and HRSID [32]) were selected to verify the effectiveness of DB-YOLO. SSDD and HRSID, including rich scenes of offshore, inshore, harbor and islands, and the specific parameters of the two data sets were shown in Table 1. The characteristics and training schemes of SSDD and HRSID data sets would be introduced as bellowing (Table 1), respectively.

(1) SSDD:

SSDD is the first open image data set for SAR ship detection (from RadarSat-2, TerraSAR-X and Sentinel-1 satellites), and it has been widely used in the study of SAR ship detection. The SSDD data set has a total of 1160 images (including 2551 labeled ships), and the image resolution ranges from 1 m to 15 m. The image size is mainly 500 × 500 pixels. In the experiments, 70% of the entire data set was randomly selected as the training set, 20% as the verifying set and 10% as the testing set.

(2) HRSID:

The images in HRSID are from Sentinel-1 and TerraSAR-X, including three single optimal resolutions of 0.5, 1, and 3 m. HRSID has a total of 5604 images (including 16,965 labeled ships) with the size of 800 × 800 pixels. Compared with SSDD, the HRSID image set owns higher resolution and richer ship characteristic information. In the experiments, HRSID used the same scheme as SSDD to generate training, verifying and testing sets.

### 3.3. Evaluation Indexes

Evaluation indexes included precision, recall, precision–recall curve (PRC) and average precision (AP). Precision represented the proportion of ships that were correctly detected in all positive detect results, and recall represented the proportion of ships that were correctly detected in the ground truth. The definition was expressed as Equations (9) and (10), respectively:

(9) $$precision=\frac{TP}{TP+FP}$$

(10) $$recall=\frac{TP}{TP+FN}$$

Average precision was calculated by using the integral area of the PRC. AP50 was the average precision calculated when IoU was 0.5, and AP was the average of the precision obtained by IoU at intervals of 0.05 from 0.5 to 0.95. The F1 score integrated precision and recall into a single indicator, evaluating the performance of the detection method comprehensively. The definition was expressed as Equations (11) and (12), respectively:

(11) $$AP=\int_{0}^{1}P\left(R\right)\cdot dR$$

(12) $$F1=2\cdot \frac{precision\cdot recall}{precision+recall}$$

### 3.4. Results and Discussion

In this section, the validity of FEN and DB-FPN modules in DB-YOLO was tested, and then the effects of DB-YOLO were compared with Faster R-CNN [11], Cascade R-CNN [38], Libra R-CNN [39], FCOS [16], CenterNet [40] and YOLOv5s on SSDD and HRSID datasets, respectively.

#### 3.4.1. Effect of FEN

In order to analyze the influence of Res blocks number in CSP blocks of layer C3–C5 in FEN on ship detection, the experiment results are shown in Table 2. As can be seen from the results, on the baseline of YOLOv5s ({3, 3, 1}), with the increase in n in the C3–C5 layers, the detection accuracy was improved. However, when the n of layer C5 increased from 1 to 3, the number of parameters increased by 22.8%, but the accuracy was almost unchanged. This was because layer C5 had the largest channel, and for small ships, the target information at the high-level feature maps was relatively vague, so it was difficult to improve the accuracy by increasing the n of the C5 layer. When n of layer C3–C4 increased from 3 to 4, the number of parameters only increased by 3.5%, but the precision increased from 63.0 to 66.5, which was much higher than the effect of increasing the n in layer C5. When n of layer C3–C4 was greater than 4, the increase in accuracy decreased with the increase in n, but the number of parameters and the amount of computation increased linearly (when n increased by 1, the number of parameters increased by 0.8 M and the FLOPS increased by 4.2 G), the cost performance of n decreased when it continued to increase thereafter. In order to maintain the balance between real-time and accuracy, {4, 4, 1} was selected as the value of n in the CSP block of the C3–C5 layer in this paper.

#### 3.4.2. Effect of DB-FPN

By comparing the effects of different feature fusion networks under the same conditions, the effectiveness of DB-FPN and the improvement effect of multi-scale ship detection were verified. In the experiment, the calculation cost and detection effect of FPN [25], PANet [26] BiFPN [27] and DB-FPN were compared, and the results were shown in Table 3. As can be seen from the results, DB-FPN improved AP by 1.1% (from 63.2% to 64.3%) compared to FPN. By adding the bottom-up path, DB-FPN better integrated the spatial information in the lower layer with rich semantic information in the deep layer. At the same time, feature multiplexing shortened the path between the layers of the same size in the network, increased the information flow between different layers, and was more conducive to the detection of ships of different scales. The effectiveness of DB-FPN was proved by contrast experiments.

#### 3.4.3. Comparison with the State-of-the-Art Methods

To verify the performance of DB-YOLO, the calculation results were compared with the other six state-of-the-art methods in the same conditions on SSDD and HRSID data sets, respectively. The two-stage detectors included Faster R-CNN [11], Cascade R-CNN [38] and Libra R-CNN [39], the single-stage detectors include FCOS [16], CenterNet [40] and YOLOv5s. The input images were fixed to 1280 × 1280 pixels, and the training procedure had 400 epochs. The experiment results are shown in Table 4. From an overall perspective, the DB-YOLO had stable performance and achieved real-time detection.

Experiment results on SSDD and HRSID showed that DB-YOLO achieved the best accuracy compared with the other six methods, which proved that it could extract powerful features to detect multi-scale ships in complex backgrounds. Compared with the baseline YOLOv5s, DB-YOLO had a significant improvement in precision (from 83.2% to 87.8% on SSDD and from 66.9% to 72.4% on HRSID). This was because FEN and DB-FPN both strengthen the sensitivity to small and medium-scale ships by redistributing the parameters and calculations of different layers, thereby reducing the false alarm rate for islands and other ship similar targets. Meanwhile, the AP of DB-YOLO was higher than other methods (64.9% on SSDD and 72.0% on HRSID), which proved that DB-YOLO could maintain high accuracy under different IoU. Among the methods tested in the experiment, YOLOv5s had the fastest detection speed (63.3 FPS), mainly because it greatly reduced the number of channels in the feature map and the repeated residual network. DB-YOLO achieved better accuracy at the cost of an increase in complexity (48.1 FPS), but it still met the requirements of real-time detection.

**Figure 7:**
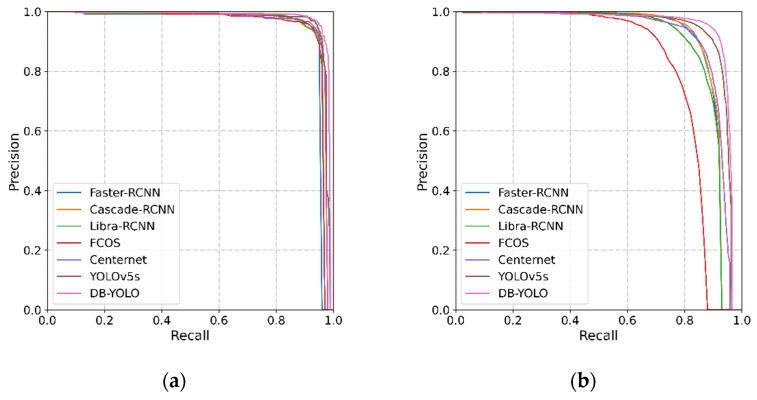
Precision–recall curves of different methods. (**a**): the experiment results on SSDD, (**b**): the experiment results on HRSID.

Figure 7 shows the precision–recall curves of different methods on SSDD and HRSID. The precision and recall were calculated at IoU = 0.5. The precision–recall curves on the two data sets both showed that DB-YOLO had higher accuracy than the other six methods.

**Figure 8:**
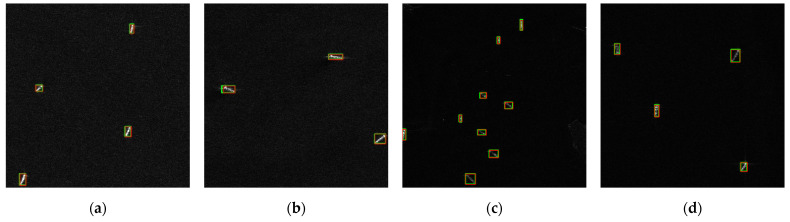

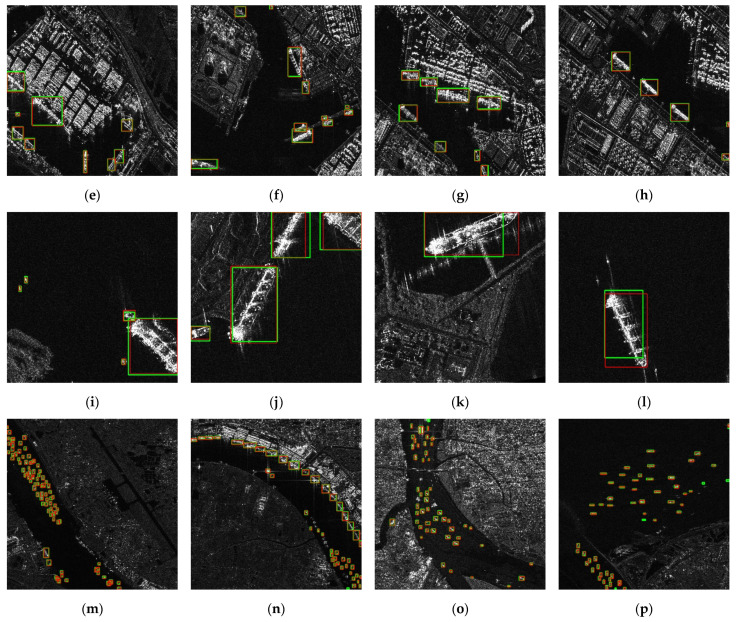
Ship detected results of DB-YOLO in HRSID. Red boxes indicate ground truth, and green boxes indicate detected ships. (**a**–**d**), (**e**–**h**), (**i**–**l**) and (**m**–**p**) in the figure showed the detection results of offshore ships, inshore ships, large-scale ships and densely distributed small-scale ships, respectively.

Figure 8 shows the visualization of DB-YOLO for detection results of offshore ships, inshore ships, large-scale ships and densely distributed small-scale ships; the red box represents ground truth, and the green box represents the detected ships. It can be seen that DB-YOLO could achieve high accuracy in both offshore (Figure 8a–d) and inshore (Figure 8e–h) situations because DB-YOLO could effectively distinguish complex backgrounds. At the same time, DB-YOLO had a relatively stable performance in the detection of large-scale ships (Figure 8i–l) and densely distributed small-scale ships (Figure 8m–p), which meant that the improved feature pyramid network had more powerful feature representation capabilities. In summary, although DB-YOLO had the problem of false alarms at the edges caused by improper image segmentation (Figure 8n–p), it could still effectively detect multi-scale ships by comparing actual ship data within the error allowable range.

#### 3.4.4. Result of DB-YOLO on Large-Scale SAR Images

**Figure 9:**
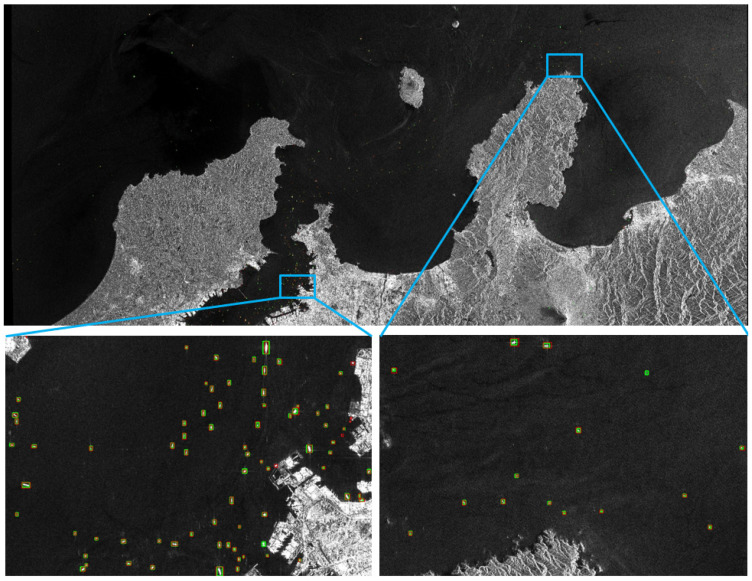
Results in large-scale SAR images. Data came from Sentinel-1. Red box indicate ground truth, and green boxed indicate predicted ships.

The robustness of DB-YOLO in real scenes was verified on large-scale images of Sentinel-1, and the results were shown in Figure 9. The SAR image came from Copernicus Open Access Hub [41], and accurate ground truth annotation was obtained through the Automatic Identification System (AIS). There was a total of 92 ships in the two enlarged images, among which 86 ships were correctly detected, 6 ships were missed and 2 ships were falsely detected (Figure 9). The precision was 97.7% and the recall was 93.5%, which proved the effectiveness and robustness of DB-YOLO in real scenes.

## 4. Conclusions

In this paper, the DB-YOLO detector was constructed to detect ships in SAR images, and it was composed of Feature Extraction Network (FEN), Duplicate Bilateral Feature Pyramid Network (DB-FPN) and Detection Network (DN). In view of the real-time requirements of SAR ship detection, a single-stage detector was proposed, and the residual network was used to improve the detection speed. In view of the requirements of multi-scale ship detection, the feature extraction capability of a lower level was strengthened by optimizing the distribution of parameters and computation of different feature layers in FEN. At the same time, DB-FPN was designed to strengthen the fusion of features and to enhance the integration of low-level spatial location information and deep semantic information. In addition, the path between the layers of the same size in the network was shortened by feature multiplexing, which was more conducive to the flow of information. Finally, experiment results of quantization and visualization on SSDD and HRSID datasets and large-scale Sentinel-1 images demonstrated the effectiveness and robustness of the DB-YOLO detector for multi-scale ship detection. Compared with the other six CNN-based methods such as Faster R-CNN, Cascade R-CNN, Libra R-CNN, FCOS, CenterNet and YOLOv5s, DB-YOLO achieved better performance.

Although DB-YOLO achieved satisfactory results in the experiment datasets, there were still false alarms at the edges of the images caused by improper image segmentation. In the future, the above problems would like to be explored and solved by optimizing the image segmentation methods and edge target detection algorithms. In view of the problem of inshore ship missing, the next research would continue to optimize the anti-jamming ability of the detector against complex environments through data argumentation.

## Figures and Tables

**Table 1:** Descriptions of existing two datasets used in experiments.

Data Sets		SSDD	HRSID
Polarization		HH, HV, VV, VH	HH, HV, VV
Image number		1160	5604
Ship number		2551	16,965
Image size (pixel)		500 × 500, etc.	800 × 800
Resolution (m)		1–15	0.5, 1, 3
Size of ships (nums)	Small	1529	9242
	Medium	935	7388
	Large	76	321

**Table 2:** Experiment of the effective of different number of Res block in CSP block.

{C3, C4, C5}	Params(M)	FLOPs(G)	P	R	F1	AP50	AP
{3, 3, 1}	22.8	52.9	63.0	91.5	74.6	91.1	63.1
{3, 3, 3}	28.0	57.1	63.0	91.7	74.7	91.4	63.1
{4, 4, 1}	23.6	57.1	66.5	91.8	77.1	91.7	63.7
{5, 5, 1}	24.4	61.3	66.8	92.0	77.5	91.8	63.8
{6, 6, 1}	25.2	65.5	67.0	92.1	77.6	91.8	63.8

**Table 3:** Experiment results of different feature fusion network.

Method	Params(M)	FLOPs(G)	P	R	F1	AP50	AP
FPN	7.2	20.8	67.8	91.1	77.8	91.6	63.2
PANet	8.3	22.1	68.4	91.3	78.2	91.9	63.6
BiFPN	8.1	21.4	68.1	91.4	78.0	91.8	63.9
DB-FPN	11.6	29.8	68.6	91.8	78.5	92.2	64.3

**Table 4:** Experiment results of different method on SSDD and HRSID. The best results were highlighted in bold.

Method	Params (M)	FLOPs (G)	Fps	SSDD					HRSID				
				P	R	F1	AP50	AP	P	R	F1	AP50	AP
Faster R-CNN	41.4	134.4	6.9	82.4	94.5	88.0	94.3	59.3	67.2	90.5	77.1	89.5	67.6
Cascade R-CNN	67.2	153.2	6.0	84.2	95.6	89.5	96.3	61.8	68.7	91.3	78.4	90.7	69.5
Libra R-CNN	42.8	141.3	6.3	83.6	94.7	88.8	94.8	59.8	66.8	89.7	76.6	88.9	67.2
FCOS	32.1	126.0	7.8	84.7	95.8	89.9	95.8	59.6	62.9	86.1	72.7	84.5	63.4
CenterNet	16.5	72.5	13.9	83.2	96.1	89.1	95.3	60.7	65.3	92.1	78.3	91.3	68.6
YOLOv5s	**7.1**	**16.4**	**63.3**	83.2	97.1	89.6	97.5	63.9	66.9	94.2	78.2	93.8	69.8
DB-YOLO	10.8	25.6	48.1	**87.8**	**97.5**	**92.4**	**97.8**	**64.9**	**72.4**	**94.9**	**82.1**	**94.4**	**72.0**

## Data Availability

No new data were created or analyzed in this study. Data sharing is not applicable to this paper.
